# Advancements and Challenges in the Preoperative Management of Pheochromocytoma in a Low- and Middle-Income Country (LMIC): A Nationwide Survey

**DOI:** 10.7759/cureus.89145

**Published:** 2025-07-31

**Authors:** Talha Asad, Muhammad D Asjad, Qandeel Farakh, Talat Waseem

**Affiliations:** 1 Surgery, Services Hospital Lahore, Lahore, PAK; 2 Surgery, Shalamar Medical and Dental College, Lahore, PAK; 3 Endocrine Surgery and Surgical Oncology, Shalamar Medical and Dental College, Lahore, PAK

**Keywords:** adrenalectomy, alpha-blockade, blood pressure optimization, catecholamine-secreting tumors, hypertension control, peri-operative management, pheochromocytoma, preoperative preparation

## Abstract

Background: Pheochromocytomas are rare catecholamine-producing neoplasms of the adrenal medulla that present considerable perioperative management challenges. Despite advances in pharmacologic protocols and surgical techniques, clinical variability, particularly in drug availability (e.g., phenoxybenzamine vs. doxazosin), lack of standardized titration protocols, and inconsistent multidisciplinary planning continue to affect management.

Objective: This study examines contemporary trends in the preoperative management of pheochromocytoma through a national survey of surgical professionals in Pakistan, aiming to uncover prevailing practices, highlight systemic gaps, and inform evidence-based refinements to current protocols.

Methods: A structured survey was disseminated to 196 practicing healthcare professionals across academic and non-academic institutions over a period of four months. Eligible participants were MBBS-qualified surgical professionals with at least one year of training and experience in the preoperative or operative management of adrenal tumors. Data were analyzed using descriptive statistics for quantitative trends and thematic analysis for qualitative feedback.

Results: Among respondents, 137/196 (69.9%) had experienced managing 1-20 pheochromocytoma cases, while 30/196 (15.3%) had operated on more than 20 cases. Blood pressure charting was one preferred preoperative evaluation method (127/196 (64.8%)), followed by confirmation through biochemical testing and localization through imaging. Selective alpha-blocker use (78/196, 39.8%) reflects both clinical preference and limited access to non-selective agents. Beta-blockers were used adjunctively by 137/196 (69.9%) to manage reflex tachycardia. Combination therapy of alpha- and beta-blockers was the predominant regimen (118/196 (60.2%)). For normotensive or alpha-blocker-intolerant patients, calcium channel blockers alone or with beta-blockers were preferred (127/196 (64.8%)). Common side effects included orthostatic hypotension (117/196 (59.7%)) and reflex tachycardia (87/196 (44.4%)). Multidisciplinary approaches involving endocrinologists and cardiologists were endorsed by 147/196 (75%). Challenges such as uncontrollable hypertension (69/196 (35.2%)) and patient non-compliance (59/196 (30.1%)) were frequently reported, with hospital admission (98/196 (50%)) and multidisciplinary collaboration (118/196 (60.2%)) used as mitigation strategies. Recent advancements such as the adoption of selective alpha-blockers, combination therapies, and invasive perioperative monitoring were associated with improved blood pressure control, reduced intraoperative instability, and enhanced overall management efficiency. The data revealed a shifting preference toward selective alpha-blockers and increased use of combination therapies. However, inconsistencies in drug titration, underutilization of structured protocols, and limited access to minimally invasive surgery remain problematic. Key qualitative themes included pharmacologic evolution, patient non-compliance, intraoperative instability, and calls for formalized training pathways.

Conclusion: The evolving landscape of pheochromocytoma management demands a more cohesive integration of pharmacological precision, multidisciplinary planning, and institutional standardization. Bridging gaps in training and access, while refining clinical protocols, will be essential to advancing safe and effective surgical outcomes.

## Introduction

Pheochromocytomas are rare neuroendocrine tumors arising from chromaffin cells of the adrenal medulla, with an estimated incidence of two to eight cases per million population per year [1]. This tumor, while uncommon, has outsized clinical significance due to its ability to secrete catecholamines, leading to labile hypertension, tachyarrhythmias, and life-threatening cardiovascular complications during surgery if not appropriately managed. Studies report perioperative hemodynamic instability in up to 50% of cases, with associated morbidity rates of 20%-30% and mortality rates historically as high as 30% in poorly controlled patients [2]. Complete surgical excision remains the mainstay of treatment; however, without optimal preoperative preparation, patients are at considerable risk of intraoperative hemodynamic instability [3].

The cornerstone of perioperative safety is effective adrenergic blockade, typically starting with alpha-adrenergic antagonists to mitigate vasoconstriction and control blood pressure. Beta-blockers are typically introduced after adequate alpha-blockade to control reflex tachycardia, thereby avoiding the risk of unopposed alpha-adrenergic stimulation [4]. Current recommendations suggest beginning pharmacologic preparation 10-14 days prior to surgery to allow for adequate dose titration and volume expansion [5].

Despite these well-accepted principles, clinical practice in preoperative management remains heterogeneous across institutions and regions, particularly in terms of alpha-blocker availability and dose titration protocols. Differences in drug availability (phenoxybenzamine vs. selective alpha1-blockers like doxazosin), anesthetic protocols, and intraoperative monitoring strategies contribute to variability in outcomes [6]. Moreover, patients in low- and middle-income countries (LMICs) face further barriers, including limited access to imaging, surgical expertise, and standardized protocols [7].

Recent literature has highlighted advancements in predictive tools for intraoperative instability, as well as novel approaches to outpatient preoperative blockade and perioperative care pathways [8,9]. Nevertheless, these practices have not been uniformly adopted, particularly in LMIC settings where context-specific data and guidelines are lacking.

To our knowledge, this is one of the first nationwide surveys from an LMIC specifically examining the preoperative management of pheochromocytoma. By capturing both quantitative trends and qualitative insights, this study addresses a critical gap in LMIC-focused endocrine surgical literature.

## Materials and methods

Study design and rationale

This study employed a cross-sectional, mixed-methods survey design to investigate contemporary practices in the preoperative management of pheochromocytoma among surgeons across Pakistan. A combination of quantitative (structured questions) and qualitative (optional open-ended responses within the same survey form) data was collected to capture not only practice trends but also the reasoning, concerns, and institutional contexts underlying them. Participants were informed that their anonymized narrative responses could be used for thematic analysis. Given the unique challenges faced in LMICs, such as inconsistent access to essential medications, limited perioperative infrastructure, and the absence of standardized national protocols, a focused, country-specific exploration of current practices is essential. This study aims to fill that gap by providing evidence on how these systemic limitations shape preoperative management strategies for pheochromocytoma in Pakistan.

Participant recruitment and sampling

A purposive sampling strategy was used to reach surgeons from academic, tertiary, and public-sector hospitals known to manage adrenal tumors. Invitations were disseminated electronically via professional surgical societies (e.g., Society of Surgeons Pakistan, Society of Oncological Surgeons in Pakistan, and Society of District Surgeons), departmental email lists, and social media forums for surgical professionals (WhatsApp and Facebook groups).

Eligibility criteria

The eligibility criteria include an MBBS qualification with at least one year of surgical training, direct or collaborative involvement in preoperative or operative care of adrenal tumors, and willingness to participate voluntarily and provide anonymous feedback.

Survey instrument development

The survey tool was developed iteratively through a Delphi-style consensus among three senior endocrine surgeons, one anesthesiologist, and one endocrinologist. Questions were designed to cover key domains reflecting the spectrum of preoperative decision-making.

The final survey instrument contained the following sections: Section A (Demographics & Experience): years in practice, surgical role, and number of pheochromocytoma cases managed; Section B (Preoperative Evaluation): methods used to assess hypertension and biochemical confirmation of diagnosis; Section C (Pharmacologic Preparation): drug choices, combination strategies, titration preferences, and rationale; Section D (Perioperative Challenges): self-reported difficulties and adverse events encountered; Section E (Multidisciplinary Planning): degree and structure of team collaboration; Section F (Training and Technological Advances): perceived preparedness and adoption of new surgical or monitoring modalities; and Section G (Open-Ended Questions): narrative input on challenges, innovations, and recommendations.

Pilot testing with six endocrine surgery fellows led to minor revisions in wording, sequencing, and dropdown standardization.

Data collection procedures

The survey was hosted on Google Forms (Google, Mountain View, CA, US) and distributed over a period of four months (October 2024-February 2025), with periodic reminders sent via email and messaging platforms to encourage participation. The details of the questionnaire are provided in the appendix. Respondents provided digital informed consent. No identifying personal information was collected to ensure privacy and candid responses. Participation was voluntary, and no incentives were offered.

Quantitative data analysis

Quantitative responses were exported into Microsoft Excel (Microsoft Corp., Redmond, WA, US) and analyzed using IBM SPSS Version 26.0 (IBM Corp., Armonk, NY, US). Descriptive statistics (frequencies, percentages, and cross-tabulations) were used to summarize responses. Key variables were stratified by the role in surgical hierarchy (consultant, registrar, and resident), number of pheochromocytoma cases managed, and institutional type (tertiary public, private academic, and district-level). Differences across stratified groups were descriptively reported; no inferential statistical tests were applied due to the exploratory design and subgroup size variability.

Qualitative data analysis

Open-ended responses were subjected to thematic analysis using Braun and Clarke’s six-step framework [10]. The following steps were taken for the analysis.

Familiarization

Responses were read and re-read to gain depth.

Initial Coding

Two independent reviewers (one endocrine surgeon and one qualitative researcher) coded the data using inductive coding.

Theme Generation

Codes were grouped into conceptual categories.

Theme Review

Emergent themes were cross-checked against original data.

Defining Themes

Five final themes were defined to encapsulate the core narratives.

Illustrative Quotations

Representative verbatim quotes were selected to reflect each theme.

Discrepancies in coding were resolved through consensus discussions. Theme clustering and matrix visualization were done.

## Results

A total of 196 surgical professionals participated in the survey, representing a wide range of designations and institutions across Pakistan. Regarding clinical experience, 29/196 (14.8%) respondents reported no prior involvement with pheochromocytoma surgeries, while a majority of 137/196 (69.9%) had operated on or been involved in one to 20 cases. A smaller subset, 30/196 (15.3%), had experience with more than 20 cases.

For preoperative hypertension evaluation, blood pressure charting was the most frequently preferred method (127/196 (64.8%)), followed by biochemical testing of plasma or urine catecholamines and metanephrines (98/196 (50.0%)), and imaging modalities such as CT or MRI (88/196 (44.9%)). Genetic testing was rarely used (8/196 (4.1%)), reflecting limited access.

In terms of pharmacologic management, non-selective alpha-blockers (e.g., phenoxybenzamine) were favored by 108/196 (55.1%) respondents, though this preference was shaped by both clinical choice and local drug availability, while selective alpha-blockers (e.g., doxazosin) were preferred by 78/196 (39.8%). Beta-blockers were used adjunctively by 137/196 (69.9%) participants to prevent reflex tachycardia. Combination therapy of alpha-blockers with beta-blockers was the most common regimen used by 118/196 (60.2%) participants, followed by alpha-blockers combined with calcium channel blockers, 49/196 (25.0%).

For normotensive patients or those intolerant to alpha-blockers, calcium channel blockers alone or with beta-blockers were preferred by 127/196 (64.8%) respondents, while beta-blockers alone were used by 39/196 (19.9%). Side effects of alpha-blockers were frequently encountered, with orthostatic hypotension reported by 117/196 (59.7%) respondents, and reflex tachycardia by 87/196 (44.4%).

Regarding clinical modalities involved in prescribing alpha-blockers, 176/196 (89.8%) emphasized medical history and physical examination, 167/196 (85.2%) used blood pressure monitoring, and 147/196 (75%) involved specialist consultations. Challenges during preoperative preparation included uncontrollable hypertension (69/196 (35.2%)), patient non-compliance (59/196 (30.1%)), and medication side effects (49/196 (25.0%)). Strategies to overcome this included hospital admission for monitoring (98/196 (50.0%)) and multidisciplinary team involvement (118/196 (60.2%)).

Finally, respondents noted significant advancements in recent years, with 137/196 (69.9%) observing a shift toward selective alpha-blockers, 118/196 (60.2%) reporting increased use of combination therapies, and 98/196 (50.0%) adopting invasive perioperative monitoring techniques. These trends reflect evolving evidence-based practices aimed at optimizing patient outcomes. Table 1 provides a summary of the quantitative data.

**Table 1:** Quantitative Data Summary

Parameter	Number (n)	Percentage (%)
Experience with pheochromocytoma surgeries		
No prior involvement	29	14.8
1–5 cases	78	39.8
6–20 cases	59	30.1
>20 cases	30	15.3
Preferred preoperative hypertension evaluation methods		
Blood pressure charting/monitoring	127	64.8
Biochemical testing (plasma/urine catecholamines/metanephrines)	98	50
Imaging (CT/MRI/MIBG)	88	44.9
Genetic testing	8	4.1
Preferred medication for blood pressure control		
Non-selective alpha-blockers (phenoxybenzamine)	108	55.1
Selective alpha-blockers (doxazosin)	78	39.8
Calcium channel blockers	10	5.1
Use of beta-blockers adjunctively		
Yes	137	69.9
No	59	30.1
Preferred drug combinations		
Alpha-blockers + beta-blockers	118	60.2
Alpha-blockers + calcium channel blockers	49	25.0
Other/monotherapy	29	14.8
Management in normotensive or alpha-blocker-intolerant patients		
Calcium channel blockers (alone or with beta-blockers)	127	64.8
Beta-blockers alone	39	19.9
Other antihypertensives	30	15.3
Common side effects of alpha-blockers		
Orthostatic hypotension	117	59.7
Reflex tachycardia	87	44.4
Dizziness/lightheadedness	81	41.3
Fatigue	50	25.5
Nasal congestion	28	14.3
Clinical modalities involved in prescribing alpha-blockers		
Medical history & physical examination	176	89.8
Blood pressure monitoring	167	85.2
Laboratory testing (catecholamines/metanephrines)	187	95.4
ECG	196	100
Specialist consultation (endocrinology/cardiology)	147	75.0
Challenges during preoperative preparation		
Uncontrollable hypertension	69	35.2
Patient non-compliance	59	30.1
Medication side effects	49	25.0
Surgical complexity	39	19.9
Postoperative complications	29	14.8
Strategies to overcome challenges		
Hospital admission for monitoring	98	50.0
Multidisciplinary team involvement	118	60.2
Medication titration/multimodal regimens	108	55.1
Observed advancements in preoperative preparation		
Shift to selective alpha-blockers	137	69.9
Increased use of combination therapy	118	60.2
Adoption of invasive perioperative monitoring	190	96.9
Use of ambulatory blood pressure monitoring	78	39.8
Enhanced multidisciplinary approaches	127	64.8

A total of 196 participants provided narrative responses. The qualitative component of this study focused on thematic analysis of open-ended survey questions, aiming to highlight the experiences and perspectives of surgeons in the preoperative management of pheochromocytoma. Dominant themes emerged, which have been highlighted in Table 2.

**Table 2:** Emerging Themes of Qualitative Analysis

Theme	Key insight	Representative quotation
Evolving Pharmacologic Strategies	Transition from non-selective to selective alpha-blockers for better tolerability and titration	“We've transitioned to using selective alpha-blockers like doxazosin due to fewer side effects compared to phenoxybenzamine.”
Hemodynamic Management Challenges	Difficulty in maintaining intraoperative BP stability despite pre-op preparation	“Intraoperative blood pressure spikes remain a significant challenge despite preoperative preparation.”
Patient Compliance and Education	Poor compliance with medication regimens necessitating better patient counseling	“Ensuring patients adhere to their medication regimen presurgery is often difficult but crucial for safety.”
Multidisciplinary Collaboration	Recognition of its value but inconsistent execution	“Close collaboration with endocrinologists and anesthesiologists is essential for successful outcomes.”
Training and Experience Gaps	Limited exposure to pheochromocytoma cases impairs surgical confidence	“Given the rarity of pheochromocytomas, more specialized training would be beneficial for surgical teams.”

The five primary themes

Evolving Pharmacologic Strategies

Surgeons highlighted a shift from non-selective to selective alpha-blockers, citing improved side effect profiles. The use and availability of the non-selective alpha blockers are the second important reason for this shift. Pharmacologically, a majority still favor non-selective alpha-blockers such as phenoxybenzamine, reflecting their established efficacy in catecholamine blockade. However, a significant and growing proportion have transitioned to selective alpha-blockers like doxazosin, likely due to their improved side effect profiles and easier titration. Beta-blockers are widely used adjunctively to mitigate reflex tachycardia, with combination therapy of alpha- and beta-blockers reported by 118/196 (60.2%) of respondents as the predominant regimen. This approach is consistent with pathophysiological principles to prevent unopposed alpha-adrenergic stimulation and cardiovascular complications. Some of the survey participants mentioned that they only utilize the selective alpha-blockers and calcium channel blockers and only reserve the beta-blockade for tachyarrhythmias alone. In normotensive or alpha-blocker-intolerant patients, calcium channel blockers alone or combined with beta-blockers are preferred by 127/196 (64.8%), indicating tailored management strategies based on individual hemodynamic status and drug tolerance.

Challenges in Hemodynamic Management

Respondents frequently mentioned difficulties in controlling intraoperative blood pressure fluctuations, emphasizing the need for vigilant monitoring. Majority of the respondents agreed that invasive monitoring of such patients is essentially required to deal with hypertensive and hypotensive crises post-excision in such patients. Orthostatic hypotension and reflex tachycardia were the most commonly encountered side effects of alpha-blockers, necessitating vigilant clinical and hemodynamic monitoring in the preoperative setting. The involvement of multidisciplinary teams-including endocrinologists, cardiologists, and anesthesiologists-was endorsed by 147/196 (75%) of respondents, highlighting the complexity of perioperative care and the need for coordinated management. Clinical modalities such as thorough medical history, physical examination, serial blood pressure monitoring, laboratory testing, and ECG were widely recognized as essential components in prescribing and titrating alpha-blockers, reinforcing the importance of individualized patient assessment.

Patient Compliance and Education

Key challenges identified include uncontrollable hypertension despite pharmacotherapy (69/196 (35.2%)) and patient non-compliance (59/197 (30.1%)), both of which significantly impact preoperative optimization. The patients often complain of blood pressure variation, and they adjust poorly to these drug-mediated blood pressure changes. Hence, they often remain either non-compliant or partially compliant, especially when they are in an ambulatory setting. Hence, non-compliance with preoperative medication regimens was a recurrent concern, suggesting the importance of thorough patient education. Respondents reported that hospital admission for close monitoring (98/196 (50%)) and multidisciplinary collaboration (118/196 (60.2%)) were effective strategies to mitigate the rapid changes in blood pressure, side effects, and non-compliance. These findings highlight systemic gaps in outpatient management and patient education, highlighting the need for structured protocols and enhanced patient engagement.

Multidisciplinary Collaboration

Surgical complexity and postoperative complications, such as hypotension and hypoglycemia, although less frequently reported, remain important issues requiring experienced surgical and anesthetic team inputs. Respondents suggested the need for national guidelines to standardize drug titration, optimize multidisciplinary workflows, and improve access to minimally invasive surgical techniques.

Training and Experience

Surgeons expressed a desire for more specialized training opportunities to enhance their competence in managing this rare tumor. Moreover, the need for more advanced laparoscopic training for adrenalectomies was also highlighted.

## Discussion

The findings from this national survey offer important insights into the contemporary preoperative practices for pheochromocytoma management in Pakistan, reflecting a broader perspective on the challenges faced in LMICs. The notable shift toward selective alpha-blockers, particularly doxazosin, aligns with global trends favoring these agents for their improved side effect profile and titratability compared to phenoxybenzamine. In the Pakistani context, the absence of national guidelines likely contributes to this shift and to broader variability in clinical practice, as choices are influenced by local availability, institutional preferences, and clinician familiarity. This mirrors observations in developed settings, where doxazosin has demonstrated similar efficacy with fewer cardiovascular side effects and lower cost implications [11].

Nevertheless, nearly half of the respondents continue to use non-selective alpha-blockers, likely due to familiarity, institutional preference, or limited availability of alternatives. Given the significant variability in preoperative management practices and the absence of national guidelines in Pakistan, there is a pressing need to develop standardized, context-sensitive protocols. Such efforts should ideally be led by a collaboration between national surgical societies, endocrinology boards, and relevant government health authorities to ensure multidisciplinary input and broad implementation.

Preoperative blood pressure control remains a central goal, yet intraoperative hemodynamic instability continues to challenge surgical outcomes. These findings are consistent with prior reports identifying preoperative hypertension severity and tumor size as key predictors of intraoperative crises [2,12]. While 118/196 (60.2%) of respondents favored combination therapy (alpha- and beta-blockade), variability in dosing protocols and titration schedules were commonly reported, reflecting a gap in standardized practices.

The use of calcium channel blockers as an alternative in normotensive or alpha-blocker-intolerant patients is supported by current evidence but should be approached cautiously. Studies have shown that while CCBs can effectively blunt blood pressure spikes, they do not prevent the alpha-mediated vasoconstriction associated with catecholamine surges during tumor manipulation [13].

A particularly compelling finding is the strong endorsement (147/196, 75%) of multidisciplinary approaches involving endocrinologists and anesthesiologists. While the survey did not differentiate between structured teams and ad hoc collaboration, the widespread support underscores a growing recognition of the value of integrated perioperative planning. This corroborates previous literature advocating for team-based care in complex adrenal surgeries [14]. However, inconsistent implementation across institutions remains a concern and points toward a need for structural reforms, including integrated preoperative conferences and perioperative planning protocols.

Patient-related factors, particularly non-compliance, were frequently cited barriers, reflecting an urgent need for better patient education, simplified medication regimens, and closer follow-up. As previous studies have noted, patient understanding of disease severity and adherence to pre-op instructions significantly affect surgical outcomes [15].

While our study primarily focused on pharmacological and institutional trends in preoperative management, it is important to highlight the underutilization of genetic testing and advanced imaging as critical areas requiring attention. Pheochromocytomas and paragangliomas exhibit a high rate of genetic predisposition, with approximately 30% of cases carrying germline mutations and up to 40% demonstrating somatic alterations. Identification of mutations, particularly in genes such as SDHB, not only informs prognosis, given their association with higher metastatic potential, but also guides surgical strategy (e.g., consideration of cortical-sparing adrenalectomy in low-risk mutations versus complete resection in high-risk genotypes). Similarly, comprehensive imaging plays an indispensable role in tumor localization, size assessment, and surgical planning. Tumor size, in particular, has shown a strong linear correlation with the risk of metastasis [16]. Despite these established benefits, only 88/196 (44.9%) of respondents in our survey reported routine use of imaging, and genetic testing was scarcely utilized, reflecting resource limitations and access disparities in LMICs. Addressing these gaps through capacity building, funding support, and integration into national protocols may significantly enhance long-term outcomes in endocrine tumor care.

Finally, limited surgical exposure and training in endocrine tumor management emerged as significant themes. With only 30/196 (15.3%) of respondents having operated on more than 20 cases, skill development programs-such as targeted fellowships, simulation-based training, and international collaborations-may be essential in improving long-term outcomes in LMICs.

To move toward safer, more standardized, and equitable care for patients undergoing surgery for pheochromocytoma, we propose the recommendations given in Table 3.

**Table 3:** Recommendations for Practice and Policy

Recommendation	Description
Institutional Standardization of Protocols	Develop clear, protocolized titration schedules for alpha- and beta-blockers based on patient risk profiles, with built-in mechanisms for dose escalation and monitoring.
Structured Multidisciplinary Management	Integrate endocrinologists, anesthesiologists, and cardiologists into formal perioperative planning pathways. Institutions should adopt tumor board-style models for adrenal pathologies.
Training and Capacity Building	Introduce simulation-based modules and workshops focused on pheochromocytoma into surgical residency programs. Utilize case-based discussions and multidisciplinary CME sessions.
Patient-Centered Education Models	Improve patient compliance and outcomes by developing educational materials on medication importance, perioperative risks, and recovery timelines, tailored to linguistic and cultural contexts.
National Data Registries & Collaborative Networks	Create multicenter pheochromocytoma registries to capture long-term outcomes, refine guidelines, and identify regional training needs.
Technological Equity	Advocate for referral pathways to centers with minimally invasive and robotic surgery capabilities, while scaling laparoscopic skills across general surgical teams.

Limitations

This study is limited by its reliance on self-reported data, potential response bias, and lack of outcome linkage to actual surgical cases. The purposive sampling method may also limit generalizability. Nonetheless, it provides a crucial snapshot of national practices and offers a foundation for future prospective studies.

## Conclusions

The preoperative management of pheochromocytoma represents a high-stakes intersection of pharmacology, surgical quality, and team-based approach. This study projects the evolving realities of clinical practice in a developing healthcare context in an LMIC. It sensitizes toward a transition in favor of more selective alpha-blockade, broader use of combination therapy, and an enhanced commitment to multidisciplinary collaboration.

Yet, this progress remains uneven. The persistence of hemodynamic crises, the variability in drug titration protocols, and the gaps in formal training reflect a landscape still in transition-defined not only by what has changed but also by what remains unresolved. Particularly striking is the gap between theoretical agreement on best practices (e.g., team-based care and preoperative planning) and the inconsistent practical implementation of these strategies.

## Appendices

**Table 4:** Questionnaire Used in the Study

Question No.	Question	Type	Options (if applicable)
1	Email	Short answer	N/A
2	Designation	Short answer	N/A
3	Institutional affiliation	Short answer	N/A
4	Which clinical records do you review during preoperative assessment?	Multiple choice (select all that apply)	-Medical history evaluation (record review) -Blood pressure charting -Other (specify)
5	What medications do you use in the preoperative period for blood pressure control?	Multiple choice (select all that apply)	-Non-selective alpha-blockers (phenoxybenzamine) -Selective alpha-blockers (doxazosin) -Calcium channel blockers (amlodipine) -Beta-blockers (metoprolol) -Other (specify)
6	How many pheochromocytoma cases have you operated on or been involved in?	Short answer	N/A
7	Preferred method for evaluating hypertension in pheochromocytoma patients preoperatively	Paragraph	N/A
8	Preferred medication for blood pressure control preoperatively	Paragraph	N/A
9	Drug combinations used for achieving effective circulatory volume and normal BP	Multiple choice	-Alpha-blockers + beta-blockers -Alpha-blockers + calcium channel blockers -Other antihypertensive drugs -Other (specify)
10	Preferred drug/combination in normotensive patients intolerant to alpha-blockers	Multiple choice	-Calcium channel blockers -Beta-blockers -Other (specify)
11	Common side effects encountered with alpha-blocker use	Multiple choice (select all that apply)	-Orthostatic hypotension -Dizziness and lightheadedness -Reflex tachycardia -All of the above -Other (specify)
12	Clinical specialties that should be involved in prescribing alpha-blockers	Multiple choice	-Cardiologist -Endocrinologist -General surgeon -All -Other (specify)
13	Have you encountered challenges during pre-op preparation? How did you overcome them?	Paragraph	N/A
14	Have you noticed changes or advancements in pre-op preparation practices over the years?	Paragraph	N/A
15	Additional information/techniques used in your setting	Paragraph	N/A
